# Demography, emergency interventions and outcome after severe pelvic injuries: a two-decade registry study from South- Western Norway

**DOI:** 10.1186/s13049-025-01399-y

**Published:** 2025-06-05

**Authors:** Kenneth Thorsen, Pieter Oord, Jon K. Narvestad, Andreas Reite, Kjell E. Tjosevik

**Affiliations:** 1 Section for Traumatology, Surgical Clinic, Stavanger University Hospital, Stavanger, Norway; 2 Department of Gastrointestinal Surgery, Stavanger University Hospital, PO Box 8100, N-4068 Stavanger, Norway; 3 Department of Clinical Medicine, University of Bergen, Bergen, Norway; 4 Department of Orthopaedic Surgery, Stavanger University Hospital, Stavanger, Norway; 5 Department of Vascular and Thoracic Surgery, Stavanger University Hospital, Stavanger, Norway; 6 Department of Emergency Medicine, Stavanger University Hospital, Stavanger, Norway

**Keywords:** Trauma, Pelvic injuries, Emergency intervention, Resuscitation

## Abstract

**Background:**

Severe pelvic injuries with ISS > 15 is associated with a high degree of morbidity and mortality. We aimed to describe the demography, emergency interventions and outcome of patients with these injuries and analyze changes in practice occurring in this timeframe.

**Methods:**

Stavanger University Hospital (SUH) is located in South-Western Norway. All patients registered in the Stavanger University Hospital Trauma registry between 2004–2022 with a pelvic injury and concomitant ISS > 15 were included in this study.

**Results:**

In total 462 patients with a pelvic injury were included in the trauma registry between 2004–2022. There were 160 patients with an ISS > 15, with 115 (72%) men and 45 (28%) women.

Median age of men was 45 years, while women were slightly older at 52 years of age. The 30-day mortality in men (25/115) and women (10/45) was identical at 22%.

Emergency intervention was performed in 33/160 (20.1%%) patients. In 27 patients a laparotomy, an EPP or an angioembolization was the primary hemostatic emergency procedure.

All but one pelvic packing were done in the years 2010–2014, with one packing also performed in 2021. A significant decline both in number of patients receiving crystalloids and the amount of crystalloids administered, both prehospitally and in the ER were seen when comparing period 1–3.

The mortality in the EPP group was very high at 6/8 (75%) while only 1/9 in the laparotomy group succumbed and 2/10 (20%) in the AE group. These discrepancies may be related to the high median ISS of 50 in the pelvic packing group, vs 40 in the angioembolization group vs 38 in the laparotomy group, reflected by a statistically significant difference in probability of survival (TRISS score) between emergency intervention groups (p < 0.001).

**Conclusion:**

Severe pelvic injuries are quite rare, with about 1 incident per month. About 2/3 are men and transport related injuries are the most common mechanism of injury. Patients in need of emergency intervention are characterized by a high median ISS and concomitant high mortality. A significant decline in emergency interventions were seen over time, which may be related to declining infusions of crystalloids and better transfusion protocols.

**Supplementary Information:**

The online version contains supplementary material available at 10.1186/s13049-025-01399-y.

## Introduction

Injuries after trauma pose a great threat to general health and life and according to the World health organization (WHO), tens of millions of people are injured each year with 4.4 million people succumbing from these injuries [1].

Pelvic injuries are common after low energy falls in elderly patients. Severe pelvic injuries, defined as a pelvic injury with an Injury severity score (ISS) > 15 are fortunately more seldom, but have been reported in up to 20% of trauma patients [2]. These injuries have a high degree of associated injuries, development of coagulopathy with life threatening bleeding and a mortality to up 40% has been reported [3–6]. The bleeding that occurs after severe pelvic fractures are due to a combination of arterial and venous bleeding from fractured bone surfaces, the sacral venous plexus and injuries to larger veins and arteries in the pelvis [6, 7].

In hemodynamic unstable patients with bleeding in the pelvis, extraperitoneal pelvic packing (EPP) and angioembolization (AE) are the treatments of choice [8]. During the latter 20 years AE has been implemented in hospitals with available radiologic resources, often as an adjunct to EPP [9]. When in extremis, emergency thoracotomies or resuscitative endovascular balloon occlusion of the Aorta (REBOA) may also be indicated for bleeding control in the exsanguinating patient [10, 11].

Severe pelvic injuries may present trauma teams with some of the most complex and demanding tasks in trauma. In this study, we aimed to describe the demography, emergency interventions and outcome of patients with severe pelvic injuries during a two-decade period and to look for changes in practice during this period of time.

## Methods

### Study population

Stavanger is located in South-Western Norway, and the twin cities of Stavanger/Sandnes account for the third densest populated city area in Norway after Oslo and Bergen.

Stavanger University Hospital (SUH) serves a population of about 400.000 people and receives trauma patients from about 600.000 inhabitants in a wider catchment area. SUH is the only hospital in this region and is after Ullevål University Hospital in Oslo, one of the busiest trauma centres in Norway. Each year about 700–800 patients are admitted for suspected or severe trauma and about one in five of the admitted patients have an ISS > 15.

The hospital has a 24/7 neurosurgical and neurointensive unit and has had a busy radiological intervention service since the early 2000 s.

The Stavanger University hospital trauma registry include all patients admitted with a trauma team activation. In addition patients admitted to the emergency room (ER) without trauma team activation but who are found to have an Injury Severity Score (ISS) > 9 on diagnostic screening or, have a penetrating injury to the head/neck/torso proximal to the elbow or knee, head injury with Abbreviated Injury Scale (AIS) ≥ 3 or ≥ 2 proximal long bone fractures are registered in the trauma registry by the trauma registrars.

All patients registered in the Stavanger University Hospital Trauma registry between 2004–2022 with a pelvic injury and concomitant ISS > 15 were included in this study.

### Definitions

Severe injuries were defined as an injury severity score (ISS) > 15 [12]. The Association for the Advancement of Automotive Medicine—Abbreviated Injury Scale 1990 revision, update 98 (AIS 98) [13] was used.

Polytrauma was defined as an AIS injury ≥ 3 in ≥ 2 regions = 2x > Ais > 2 in accordance with the Newcastle definition [14].

Location of major injury (LOMI) was defined as the injured region with highest AIS. If two regions had equally high AIS, but one region had an additional injury with higher AIS, then the region with the highest total trauma load was classified as LOMI.

In a few cases a patient had more than one LOMI.

### Time periods

2004–2008 were classified as period 1, 2009–2014 period 2 and 2015–2022 period 3.

### Study design

This observational cohort study is a retrospective analysis of a prospectively maintained institutional trauma registry database, covering all trauma patients admitted to Stavanger University Hospital between January 1 st, 2004, and December 31 st, 2022.

The study was reported according to the STROBE guidelines [15].

### Ethics

This study was approved by the local data protection officer.

## Results

### Demographics

A total of 9690 patients were included in the trauma registry between 2004–2022 and 2071 patients had an ISS > 15.

A pelvic injury was registered in 462 patients and 160 of them had an ISS > 15. Men dominated with 115 (72%) and 45 (28%) were women.

Further baseline characteristics are presented in Table 1.

**Table 1:** Characteristics of patients with severe pelvic trauma treated between 2004–2022

*N* = 160	Males	Females	*P* value
Age	45	52	0.38
ISS	34	34	0.81
Systolic blood pressure	116	125	0.19
Pulse	92	87	0.50
RR	22	22	0.45
ASA Score			0.098
1	72	20	
2	28	15	
3	23	10	
4	2	0	
Polytrauma	99/115 (86%)	40/45 (89%	0.64
LOMI			0.81
Head	14	6	
Neck/spine	1	0	
Thorax	41	18	
Abdomen	8	1	
Pelvis	54	22	
Extremity	1	0	
LOMI 2			0.47
Head			
Neck/spine	1		
Thorax	5		
Abdomen	5	4	
Pelvis	19	8	
Extremity	3	1	
TRISS	0.88	0.80	0.38
Mortality	25/115 (22%)	10/45 (22%)	0.95
Mechanism of injury			
Transport related	68/115 (59%)	26/45 (58%)	0.88
Fall	38/115 (33%)	16/45 (36%)	0.76
Alcohol	15/115 (13%)	2/45 (4%)	0.28

Age, Blood pressure, pulse, respiration rate (RR) and Iss presented as median values

Median age of men was 45 years, while women were slightly older at 52 years of age. All 160 patients had a blunt injury mechanism. Transport related injuries were most common with 94/160 (59%) followed by falls (54/160 (34%)). Transport related injuries were most commonly encountered in patients aged < 60 years, while for patients aged ≥ 60 years falls and transport related injuries were equally common.

Transport to hospital was done by ambulance in 83 patients, by helicopter in 75 patients, by a bystander in one case and unknown in one case.

The accidents occurred in 9 different counties with a total of 26 patients injured in counties other than the primary catchment area.

No patients were registered with hypothermia and 17/160 (10%) patients tested positive for ethanol.

### Diagnostics

A pelvic x-ray was taken in the trauma bay in 128/160 (80%) patients, with 112 pathological and 21 described as normal. Of these 21 normal x rays, 18 patients went to a CT scan with pathologic findings.

A CT scan including the pelvis was taken in 135/160 (84%) patients with all showing signs of pathology.

Emergency intervention was performed in 33/160 (20%) patients (Table 2). In 27 patients a laparotomy, an EPP or an AE was the primary hemostatic emergency procedure. One patient had an emergency thoracotomy, two patients with severe head injuries had an intracranial pressure monitor and 3 patients had other interventions not specified in the trauma registry.

**Table 2:** Comparison of injury severity, location of major injury and outcome according to primary hemostatic emergency intervention

*N* = 27	Laparotomies *N* = 9	Extra peritoneal packing *N* = 8	Angioembolization *N* = 10	*P* value
Male	8	7	7	*P* = 0.5
Female	1	1	3	
ISS median	38	50	40	*P* = 0.044
NISS median	43	53	48	*P* = 0.15
TRISS score median	0.87	0.52	0.81	*P* = 0.105
RTS Median	6.9	6.0	7.6	*P* = 0.056
Mortality	1/9 (11%)	6/8 75%	2/10 (20%)	*P* = 0.011
Systolic BP < 90 in ED	3/9	1/8	2/10	*P* = 0.39
Pulse > 90	6/9	6/8	8/10	*P* = 0.287
Tile (A-C)				*P* = 0.316
A1	1	0	0	
A2	0	0	1	
B1	3	2	0	
B2	3	5	3	
B3	1	0	1	
C1	1	2	3	
C2	0	1	0	
Crystalloids administered in ER	2100	2375	2000	*P* = 0.241
Blood transfusion in ED	9/9	8/8	5/10	*P* = 0.022
Number of transfusions in ED	3	3	1	*P* = 0.048
LOMI				*P* = 0.506
Pelvis	1	6	3	
Thorax	5	1	6	
Head	0	1	0	
Abdomen	3	0	1	
LOMI2				*P* = 0.311
Pelvis	2	2	5	
Thorax	1	0	0	
Head	0	0	0	
Abdomen	0	0	0	
ICU days	3	2	2	*P* = 0.270
LOS	7	3.5	4.5	*P* = 0.363

Ml of crystalloids and units of blood administered in ER pre

All but one EPP were done in the years 2010–2014, with one EPP also performed in 2021. The mortality in the EPP group was very high at 6/8 (75%) while only 1/9 in the laparotomy group succumbed and 2/10 (20%) in the AE group.

Angioembolization was similarly distributed over time as EPP with all but one procedure done in the years 2009–2014, with one procedure in 2017.

Comparison of injury severity, treatment and outcome for the three time periods are presented in Table 3.

**Table 3:** Comparison of injury severity, treatment and outcome in period 1–3

*N* = 160	Period 1	Period 2	Period 3	*p*-value
ISS median	34	34	34	*p* = 0.673
TRISS score median	0.86	0.85	0.91	*p* = 0.603
RTS Median	7.3	7.1	7.6	*p* = 0.982
Mortality	12/68 (17.6%)	12/47 (25.5%)	11/45 (24.4%)	*p* = 0.534
Number of patients receiving crystalloids prehospitally	40/68 (58.8%)	30/47 (63.8%)	15/45 (33.3%)	*p* = 0.004
Median ml crystalloids adm. prehosp	1000	1000	750	*p* = 0.004
Number of patients receiving crystalloids in ER	60/68 (88.2%)	46/47 (97.9%)	28/45 (62.2%)	*p* < 0.001
Median ml Of Crystalloids in ER	2000	2000	1000	*p* < 0.001
Number of patients receiving blood transfusion in ER	34/68 (50.0%)	21/47 (44.7%)	16/45 (35.6%)	*p* = 0.370
Blood transfusion in ED	7.5	4	5.5	*p* = 0.370
ICU days median	3	2	2	*p* = 0.270
LOS median	7	3.5	4.5	*p* = 0.363
Emergency procedure				*p* < 0.001
Laparotomy	0	5	4	
EPP	0	7	1	
AE	3	6	1	

ISS, TRISS, RTS, ml crystalloids, units of blood transfused in ER, ICU days and LOS are all give as median values

### Multiple emergency procedures

Fourteen of the patients who had an emergency procedure, had more than one procedure done.

The most common combination was a laparotomy + EPP, which was seen in nine patients, where 2 of these patients also had an adjunct AE.

One of the patients who had an initial laparotomy also had an AE.

Two patients had a laparotomy, EPP and thoracotomy, and one of these also an adjunct AE.

One patient had a thoracotomy followed by an EPP in the emergency room.

Five of the patients who had a laparotomy had ongoing abdominal bleeding that were stopped with hemostatic sutures and packing.

In total 3 patients had a thoracotomy as part of the emergency hemostatic surgery.

### Crystalloids and transfusions

**Fig. 1:**
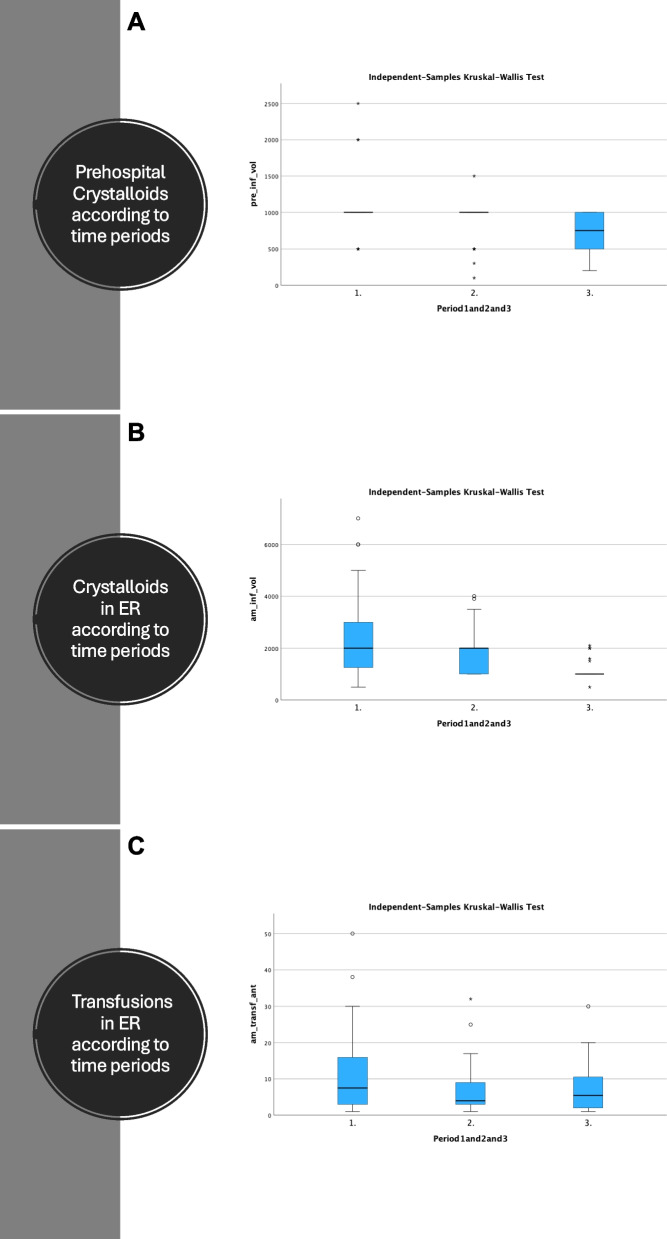
Panel **A **Prehospital crystalloids according to time periods. **B** Crystalloids administered in ER according to time periods. **C** Transfusions in ER according to time periods

A statistically significant decline in frequency and amount of crystalloids given were seen from period 1 to 3, while the amount of blood transfusions administered in the ER remained stable over time (Fig. 1 panel A-C).

### Mortality

No difference in mortality was seen between the three time periods.

**Fig. 2:**
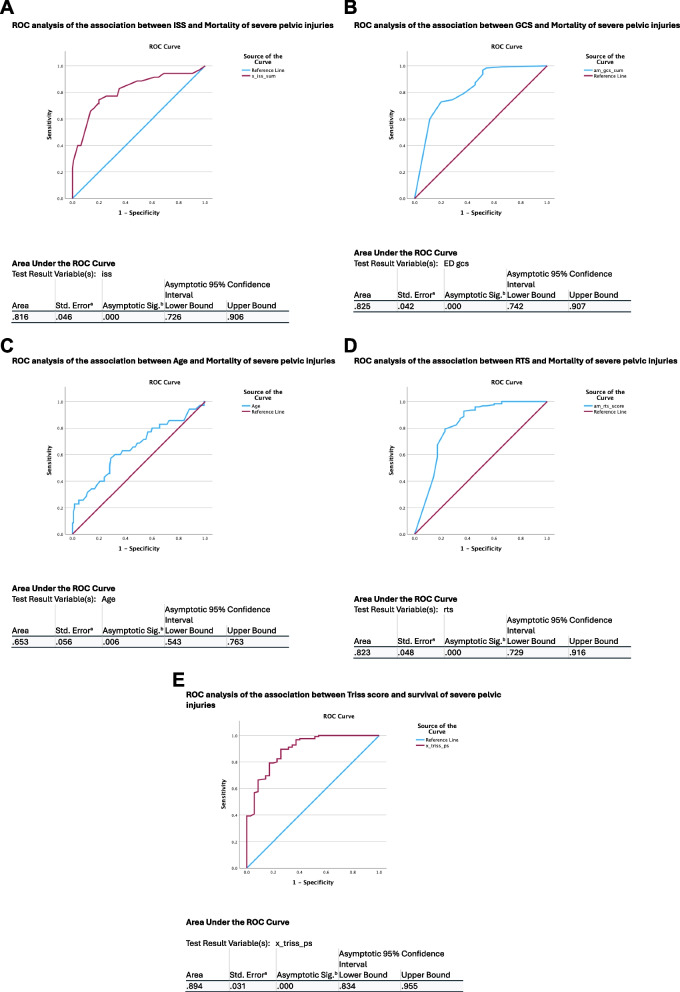
Panel **A** ROC analysis of the association between ISS and Mortality of severe pelvic injuries. Panel **B** ROC analysis of the association between GCS and Mortality of severe pelvic injuries. Panel **C** ROC analysis of the association between Age and Mortality of severe pelvic injuries. Panel **D** ROC analysis of the association between RTS and Mortality of severe pelvic injuries. **E** ROC analysis of the association between Triss score and survival of severe pelvic injuries

A cut off point of 35 years of age had a sensitivity of 80% for mortality by ROC analysis. Figure 2 panel A-E shows ROC curves and area under the curve for factors associated with 30-day mortality.

The mortality increased from 13% in those aged 35 and younger to 26% in those aged > 35 years of age. Further, the mortality increased for each decade to 32% for those aged > 50, 35% in those aged > 60, 43% in those aged > 70 and 67% in those aged > 80 years of age.

Three patients were > 90 years of age and they all died.

Univariate analysis and multivariable regression analysis of factors associated with mortality is presented in table S1 and table S2 (supplementary).

A TRISS score < 0.22 had a sensitivity of 0.98 for 30-day mortality by ROC analysis with an area under the curve of 0.91 (figure).

Causes of death are presented in Table 4, where 3 deaths occurred after 30 days.

**Table 4:** Cause of death in patients with severe pelvic injuries according to time periods

Cause of death	Period 1	Period 2	Period 3	Number of patients
Asphyxia	0	0	1	1
Bleeding in abdomen	1	0	0	1
Bleeding pelvis	1	0	0	1
Bleeding multiple sites	4	4	7	15
Bleeding thorax	1	0	0	1
MOF	3	4	3	10
Head injury	3	1	4	8
Other	0	1	1	2
Total	13	10	16	39

*MOF* Multiorgan failure

Only one death occurred in patients without polytrauma.

## Discussion

Severe pelvic injuries are quite rare with about one incident a month in this region. These patients are prone to polytrauma with a concomitant high mortality, especially in the elderly. Emergency hemostatic procedures were rare in the first period, most frequent in the second period, before significantly declining in period 3. A significant decline both in number of patients receiving crystalloids and the amount of crystalloids administered, both prehospitally and in the ER were seen over time. There are several possible explanations for this pattern. Firstly, in the early 2000 s a proper trauma system was not in place in Norway, nor in this region. During the second time period, ATLS principles were implemented and from 2009 several changes occurred in our trauma system including a massive transfusion protocol, weekly simulation and structured training of emergency procedures on cadavers by trauma team leaders, a practice still ongoing today [16].

Infusion of crystalloids was controversial for a long time but is now a recognized cause of dilutional coagulopathy [17, 18]. The results from this study suggest an effect from reducing the amount and frequency of administration of crystalloids both prehospitally and in the ER to the reduction of emergency procedures done in period 3. There were no significant different findings regarding blood transfusion administered in the ER during the three time periods. However, we do not have information about the amount and components transfused after leaving the ER. Since implementing massive transfusion protocols, much more focus have been given to correcting coagulopathy, preventing dilution and transfusing the bleeding trauma patients in an optimal way. Hence, we suspect that better transfusion plays a significant role in the declining need for emergency intervention during this time span.

Thus, the more restrictive use of crystalloids in combination with better transfusion protocols may have led to emergency hemostatic surgery for severe pelvic injuries decreasing in period 3, with only 6 patients receiving such. A similar development was also seen in a study from Oslo [19].

A possible influence on the peak of emergency interventions in the second period could also be attributed to the increased focus on ATLS principles and training of emergency procedures during period 2. A rising focus on training and simulation for the trauma team members may have led to initiate emergency interventions with a lower threshold. However, this notion remains speculation.

The mortality in the EPP group was especially high at 75% (6/8) while only 1/9 in the laparotomy group succumbed and 2/10 in the AE group. These discrepancies may be related to the high median ISS of 50 in the pelvic packing group, vs 40 in the angioembolization group vs 38 in the laparotomy group, reflected by a statistically significant difference in probability of survival between emergency intervention groups. However, a recent study attributed higher mortality in the EPP group to the procedure itself including a higher risk of venous thromboembolism after this procedure [20]. Nevertheless, the number of patients with an EPP in this study are small and difficult to conclude from.

The predictors of mortality by a multivariable regression model were in this study found to be a combination of ISS ≥ 28, age ≥ 36, RTS ≤ 6, GCS ≤ 9 and ASA score. These factors obviously represent a worsening of the patient’s physiology, anatomic injury severity, increasing degree of comorbidity and increasing age. Increasing mortality has been found to be associated with increasing age in pelvic trauma also by others [10, 21].

Data from the national trauma registry in Norway from 2023 also show that deaths from trauma in general is relatively higher for patients aged > 60 years of age [22].

The probability of survival represented by TRISS score was low in patients who succumb, meaning they have statistically poor odds for survival. To some extent, this could be seen as a surrogate marker for quality of treatment.

The reducing frequency of emergency intervention may also pose a problem for the trauma teams, which must now rely on training and simulation, with very few real-life cases handled by each team member. However, training and simulation have been identified as valid adjuncts to actual surgical procedures in reaching and maintaining proficiency [23]. The sickest trauma patients may still be out of reach to rescue, but only continued focus on improvement despite few real-life cases are of vital importance to maintain a high-quality trauma system.

### Limitations

The study is retrospective with the inherent weakness of such studies. We lack information about transfusions and administration of fluids post emergency department. However, the information registered up to that time point is valid. In addition, Stavanger University Hospital is the only hospital in this region and as such we present a representative study population.

## Conclusion

Severe pelvic injuries are quite rare, with about 1 incident per month. About 2/3 are men and transport related injuries are the most common mechanism of injury. Patients in need of emergency intervention are characterized by a high median ISS and concomitant high mortality. A significant decline in emergency interventions were seen over time, which could be related to declining infusions of crystalloids and better transfusion protocols.

## Supplementary Information

Supplementary Material 1.

Supplementary Material 2.

## Data Availability

No datasets were generated or analysed during the current study.
