# Correction: Demography, emergency interventions and outcome after severe pelvic injuries: a two-decade registry study from South- Western Norway

**DOI:** 10.1186/s13049-025-01436-w

**Published:** 2025-07-14

**Authors:** Kenneth Thorsen, Pieter Oord, Jon K. Narvestad, Andreas Reite, Kjell E. Tjosevik

**Affiliations:** 1 Section for Traumatology, Surgical Clinic, Stavanger University Hospital, Stavanger, Norway; 2 Department of Gastrointestinal Surgery, Stavanger University Hospital, PO Box 8100, N-4068 Stavanger, Norway; 3 Department of Clinical Medicine, University of Bergen, Bergen, Norway; 4 Department of Orthopaedic Surgery, Stavanger University Hospital, Stavanger, Norway; 5 Department of Vascular and Thoracic Surgery, Stavanger University Hospital, Stavanger, Norway; 6 Department of Emergency Medicine, Stavanger University Hospital, Stavanger, Norway

**Correction: Scand J Trauma Resusc Emerg Med (2025) 33:102**

10.1186/s13049-025-01399-y

Following the publication of the original article, the authors reported that the Abstract and Results section incorrectly stated that 2,283 patients with pelvic injuries were registered in the trauma registry during the study period. The correct number is 462 patients.

The authors clarified that this error does not affect the analysis or conclusions of the study, as the article only included patients with severe pelvic injuries (160 patients).

The original article has been updated accordingly.

