# Supplementary material for: Demography, emergency interventions and outcome after severe pelvic injuries: a two-decade registry study from South- Western Norway
Source: Scand J Trauma Resusc Emerg Med. 2025 Jun 5;33:102. doi: 10.1186/s13049-025-01399-y (PMC12142862; doi:10.1186/s13049-025-01399-y)
Supplement: Supplementary file 1 — Supplementary Material 1. [file 13049_2025_1399_MOESM1_ESM.docx]

**Table S1**

**Univariate analysis of factors associated with mortality**

| **Variable** | **P value** |
| --- | --- |
| Sex | 0.947 |
| ED BP<90 | 0.82 |
| ED Pulse>90 | 0.676 |
| RTS≤6 | <0.001 |
| GCS≤9 | <0.001 |
| Age≥ 36 years | 0.029 |
| Polytrauma | 0.042 |
| LOMI head | 0.012 |
| ISS≥ 28 | <0.001 |
| Emergency intervention type | <0.011 |
| Fall injury | 0.742 |
| Transport injury | 0.544 |
| Weekday | 0.126 |
| Month | 0.777 |
| Year | 0.504 |

Figure Legend

LOMI= location of major injury

GCS= Glasgow coma scale

RTS= Revised trauma score

BP= Systolic blood pressure
