# Supplementary material for: Demography, emergency interventions and outcome after severe pelvic injuries: a two-decade registry study from South- Western Norway
Source: Scand J Trauma Resusc Emerg Med. 2025 Jun 5;33:102. doi: 10.1186/s13049-025-01399-y (PMC12142862; doi:10.1186/s13049-025-01399-y)
Supplement: Supplementary file 2 — Supplementary Material 2. [file 13049_2025_1399_MOESM2_ESM.docx]

**Table S2 Multivariable logistic regression analysis of factors associated with mortality**

| **Variables in the Equation** | | | | | | | |
| --- | --- | --- | --- | --- | --- | --- | --- |
|  | | B | S.E. | Wald | df | Sig. | Exp(B) |
| Step 1^a^ | RTS6 | -3.418 | .677 | 25.493 | 1 | <.001 | .033 |
|  | Constant | 1.808 | .241 | 56.187 | 1 | <.001 | 6.100 |
| Step 2^b^ | RTS6 | -3.590 | .724 | 24.608 | 1 | <.001 | .028 |
|  | x_asa_score_pre |  |  | 9.778 | 3 | .021 |  |
|  | x_asa_score_pre(1) | .715 | .650 | 1.210 | 1 | .271 | 2.044 |
|  | x_asa_score_pre(2) | -1.334 | .576 | 5.361 | 1 | .021 | .263 |
|  | x_asa_score_pre(3) | -1.966 | 1.451 | 1.836 | 1 | .175 | .140 |
|  | Constant | 1.320 | .415 | 10.133 | 1 | .001 | 3.742 |
| Step 3^c^ | RTS6 | -2.924 | .778 | 14.111 | 1 | <.001 | .054 |
|  | GCS9 | -1.241 | .553 | 5.039 | 1 | .025 | .289 |
|  | x_asa_score_pre |  |  | 9.720 | 3 | .021 |  |
|  | x_asa_score_pre(1) | .734 | .661 | 1.232 | 1 | .267 | 2.083 |
|  | x_asa_score_pre(2) | -1.335 | .595 | 5.040 | 1 | .025 | .263 |
|  | x_asa_score_pre(3) | -2.212 | 1.459 | 2.298 | 1 | .130 | .110 |
|  | Constant | 1.508 | .430 | 12.291 | 1 | <.001 | 4.520 |
| Step 4^d^ | Age36 | -1.489 | .654 | 5.181 | 1 | .023 | .226 |
|  | RTS6 | -3.133 | .836 | 14.057 | 1 | <.001 | .044 |
|  | GCS9 | -1.514 | .589 | 6.616 | 1 | .010 | .220 |
|  | x_asa_score_pre |  |  | 6.926 | 3 | .074 |  |
|  | x_asa_score_pre(1) | 1.001 | .675 | 2.198 | 1 | .138 | 2.720 |
|  | x_asa_score_pre(2) | -.873 | .623 | 1.967 | 1 | .161 | .418 |
|  | x_asa_score_pre(3) | -1.725 | 1.469 | 1.379 | 1 | .240 | .178 |
|  | Constant | 2.814 | .746 | 14.236 | 1 | <.001 | 16.679 |
| Step 5^e^ | ISS28 | -1.475 | .714 | 4.266 | 1 | .039 | .229 |
|  | Age36 | -1.593 | .656 | 5.889 | 1 | .015 | .203 |
|  | RTS6 | -2.946 | .843 | 12.223 | 1 | <.001 | .053 |
|  | GCS9 | -1.250 | .594 | 4.432 | 1 | .035 | .287 |
|  | x_asa_score_pre |  |  | 7.590 | 3 | .055 |  |
|  | x_asa_score_pre(1) | 1.016 | .683 | 2.211 | 1 | .137 | 2.762 |
|  | x_asa_score_pre(2) | -.982 | .645 | 2.316 | 1 | .128 | .375 |
|  | x_asa_score_pre(3) | -2.029 | 1.583 | 1.644 | 1 | .200 | .131 |
|  | Constant | 3.860 | .940 | 16.850 | 1 | <.001 | 47.486 |
| a. Variable(s) entered on step 1: RTS6. | | | | | | | |
| b. Variable(s) entered on step 2: x_asa_score_pre. | | | | | | | |
| c. Variable(s) entered on step 3: GCS9. | | | | | | | |
| d. Variable(s) entered on step 4: Age36. | | | | | | | |
| e. Variable(s) entered on step 5: ISS28. | | | | | | | |
